# Nonsurgical approach for strip perforation repair using mineral trioxide aggregate

**DOI:** 10.4103/0972-0707.66721

**Published:** 2010

**Authors:** Savitha Adiga, Ida Ataide, Marina Fernandes, Subhash Adiga

**Affiliations:** Department of Conservative Dentistry and Endodontics, Goa Dental College and Hospital, Bambolim, Goa - 403 202, India; 1 Department of Oral Medicine and Radiology, RV Dental College, Bangalore - 560 078, India

**Keywords:** Mineral trioxide aggregate, root perforation, strip perforation

## Abstract

“Stripping” is lateral perforation is caused by overinstrumentation through a thin wall in the root and is most likely to occur at the inside wall of a curved canal, such as the distal wall of the mesial roots in mandibular first molars. In the past, poor prognosis for strip and furcation perforations was probably due to bacterial leakage or lack of biocompatibility of repair materials. However, the recent development in the techniques and materials such as mineral trioxide aggregate (MTA) has enhanced the prognosis for such cases. There is limited literature on use of MTA as an obturating material in the treatment of strip perforation. This study presents two cases of strip perforation that are successfully repaired nonsurgically using MTA with 2-year follow up. Cases suggest that MTA can be used as an alternative root canal obturation material for the treatment of strip perforation. The property differences between gray and white MTA are reviewed.

## INTRODUCTION

Although caries or resorptive processes may cause perforations, most root perforations are induced iatrogenically (zip, strip, and furcal perforations).[1] Various prognostic indicators influence the outcome of perforated teeth.[2] Perforations that are fresh, small, and those occurring coronal or apical to the crestal bone and attachment apparatus, which can be repaired immediately, have good treatment prognosis.[2,3] When left untreated, perforations close to the crestal bone and epithelial attachment or on the floor of the pulp chamber have the worst prognosis.

Several materials such as amalgam, zinc oxide eugenol, IRM, glass ionomer cement, calcium hydroxide and mineral trioxide aggregate (MTA) have been documented to be used as sealers in root perforations.[2] MTA was introduced by Torabinejad *et al*.[4] and has been recommended as a repair material for root perforations. Different leakage approaches such as fluid filtration technique,[5] dye-leakage model,[6] and bacterial leakage model[7] have proved that MTA showed better sealing ability than other materials. MTA has a high pH (12.5), antimicrobial properties, and excellent biocompatibility, which promotes the growth of cementum and formation of bone, which in turn leads to the regeneration of the periodontal ligament around the site of injury.[8] In a human osteoblast model, Koch *et al*. found that MTA stimulated the upregulation of cytokines, such as interleukin (IL)-1α, IL-1β, and IL-6, which are involved in bone turnover.[9] All these properties make the MTA a material of choice for repairing root perforations. However, the elevated cost and delayed setting time (approximately 3 h) of this product has not allowed its use in all the levels of dental health care. Recently, MTA-Angelus has been introduced as a competitor to ProRoot MTA.[10] MTA-Angelus is found to have good handling characteristics in addition to faster setting time, and it is cost effective. There is limited literature on the use of MTA-Angelus as an obturating material in the treatment of strip perforation. The purpose of this article is to present two cases of strip perforations repaired using MTA-Angelus and followed up for 2 years to evaluate the prognosis.

## CASE REPORTS

### Case 1

**Figure 1:**
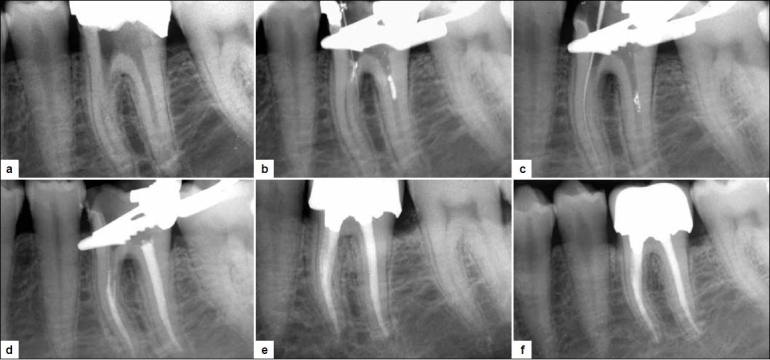
a) Pre-operative radiograph showing radio-opaque filling material coronally with loss of continuity of lamina duer and radiolucency at the mesial furcation with widening of periodontal ligament space in mesial root apex. b) Detection of strip perforation with no. 08 K-file in the mesiobuccal canal. c) Patency of the mesial canals established. d) Mineral trioxide aggregate obturation in mesiobuccal canal till the orifice; ML and Distal canals were obturated simultaneously. e) On follow up at 6 months, furcal bone formation is evident. f) In the 2-year follow-up radiograph, osseous regeneration is observed at the apex and furcation; normal periodontal ligament space is observed

A 38-year-old female presented with the strip perforation in the mesiobuccal canal in the lower left first molar, which occurred during the endodontic treatment by an undergraduate student. Her medical history was non-contributory. Her dental history revealed that the tooth had been treated by a general dentist 3 months earlier. On oral examination, there was an amalgam filling in tooth no. 19. There were no probing defects or sinus tract. The tooth was sensitive to percussion and palpation. Radiographic examination of tooth no. 19 revealed radio-opaque restorative material in the occlusal third of the crown without extending into the pulp chamber and radiolucency in the pulp chamber suggestive of cotton pellet. Root canals revealed lack of any radio-opaque obturating material, break in the continuity of lamina dura near mesial furcation, furcation radiolucency and widening of periodontal ligament space (PDL) at the mesial root apex [Figure 1a]. Provisional diagnosis of pulp necrosis with chronic apical periodontitis was established.

After isolating the tooth with rubber dam, amalgam filling was removed. Canal orifices were located. While negotiating the mesiobuccal canal with no. 15 K-file, a sudden occurrence of hemorrhage in the canal was noted; this was a suspicious sign of perforation. Periapical radiograph was taken by placing no. 08 K file in the mesiobuccal canal, confirming the strip perforation [Figure 1b]. Hemorrhage was controlled by irrigating the canal with 3% sodium hypochlorite (NaOCl), and the area was dried with gelatin sponge (Ab Gel; Sri Gopal Krishna Labs, Mumbai, India). Then, the mesiobuccal canal was located with no. 15 K-file. All the canals were cleaned and shaped with rotary ProTapers using the crown down approach. MTA powder was mixed with distilled water in the ratio 1:1 till wet sand consistency was attained. MTA-Angelus was taken with a spoon excavator and then condensed with a plugger. The mesiobuccal canal with the perforation was obturated with MTA-Angelus from the apex up to the orifice region. Then, the mesio-lingual and distal canals were obturated with Gutta-percha (GP) and AH-plus sealer [Figure 1c, d]. On follow up after 15 days, the patient was asymptomatic. At the subsequent visit, the tooth was restored with composite and porcelain fused to metal crown was placed. On follow up after 6 months, radiographic evidence of bone formation adjacent to the MTA was obtained [Figure 1e]. On follow up after 2 years, a complete resolution of furcal and peri-apical radiolucency was observed [Figure 1f].

### Case 2

**Figure 2:**
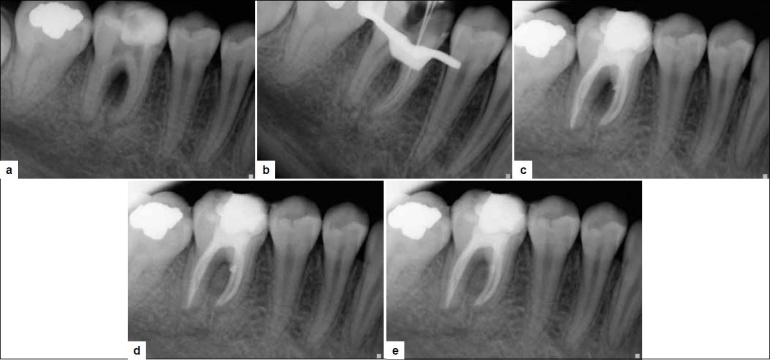
a) Pre-operative radiograph showing large furcal radiolucency and apical radiolucency at the mesial and distal roots. b) Mesiobuccal and mesiolingual canal location with no. 15K-file. c) MTA condensed into the perforation site; slight extrusion of MTA near distal aspect. d) 6 months follow up, decreased radiolucency and osseous healing at the furcation. e) 2 years follow-up

A 42-year-old male was referred to the department with a chief complaint of mild pain and tenderness to biting pressure in the right lower posterior region. Dental history revealed an attempted root canal treatment by a general dentist 6 months before. On examination, the mandibular right molar was found to have a temporary filling material. The tooth was tender to percussion and had a draining sinus tract on the buccal mucosa. There was a localized 6 mm deep periodontal pocket adjacent to the buccal furcation. Radiographic examination revealed independent radiolucent lesions at the apex of the mesial root and in the furcation region [Figure 2a].

The presence of the periodontal pocket and associated furcal destruction were suspicious signs of perforation at the mesiobuccal root of tooth no. 30. The treatment of perforation with MTA-Angelus was planned. After the patient was administered with local anesthetic, the temporary filling material was removed from the pulp chamber revealing profuse bleeding from the chamber. The chamber was irrigated with 3% NaOCl, and bleeding was controlled. Canals were dried with paper point. Hemorrhage was located along the side of the paper point in the mesiobuccal canal, which was suggestive of strip perforation. The mesiobuccal and mesiolingual canals were located with no. 15 K file [Figure 2b]. All the canals were cleaned and shaped with rotary ProTapers using the crown down approach, and the master cones were selected. Next, calcium hydroxide was packed in the canal, and access was sealed with intermediate restorative material (IRM). After 1 week, the canals were irrigated with 3% NaOCl and then dried with paper points. The mesiobuccal canal was obturated with ProTaper GP till the strip perforation site. Gray MTA-Angelus was applied in a manner similar to that described for case 1. There was slight extrusion of the material into the site [Figure 2c]. The mesiolingual canal and distal canal were then obturated with GP and AH-plus sealer. Then, the access was sealed with IRM. On follow up after 4 weeks, the sinus tract had completely healed and the access was filled with composite. On follow up after 6 months, radiographic evidence of bone formation adjacent to the MTA and reduced probing depth (3 mm) was obtained [Figure 2d]. 2-year follow up showed complete osseous healing at the apex and furcation [Figure 2e].

## DISCUSSION

Cross section of the midroot areas of curved mesiobuccal root of molars reveal that the bulkiest portion of the root structure (safety zone) lies away from the direction of the root curvature. The thin inner wall or concave wall (danger zone) lies in the direction of curvature, such as distal wall of mesial roots in mandibular first molars.[11] Stripping refers to a thinning of this concave wall or lateral wall with eventual perforation, which is caused by overzealous instrumentation. Perforations can also be caused by the inability to maintain canal curvature because of ledge formation. Furcal and strip perforations are followed by bacterial contamination, periradicular tissue injury, inflammation, bone resorption, periodontal fiber destruction, epithelium proliferation, and periodontal pocket development.[2] The principle goal of endodontic therapy is to remove bacteria and seal the root canal to promote osseous regeneration.[8]

Two major brands of MTA are available on the market: MTAAngelus (used in the case reports described here) and Pro-Root MTA (Maillefer, DENTSPLY, Switzerland). Both products are available in gray or white forms. According to the manufacturer’s material safety data sheet, the composition of ProRoot MTA is 75% Portland cement, 20% bismuth oxide and 5% dehydrated calcium sulfate, whereas that of MTAAngelus is 80% Portland cement and 20% bismuth oxide with no calcium sulfate.[12]

The dominant compounds in both types of Pro-Root MTA are calcium oxide, silica and bismuth. However, the gray version has greater concentrations of aluminum oxide (122% higher), magnesium (130% higher) and especially iron (1000% higher).[13] Although both the gray and the white versions of ProRoot MTA perform similarly in terms of furcal sealing[14] and antimicrobial effectiveness,[15] the gray version has a more favorable behavior *in vitro* in terms of development of odontoblasts,[16] whereas the white version is associated with the development of cementoblasts and keratinocytes.[17] The white version eliminated the grayness of the original MTA, which can create a shadow under a thin tissue.[14] There are certain similarities in properties between white and gray versions of MTA-Angelus and ProRoot MTA: pH 9 after 168 h,[18] overall composition,[12] cytological investigation, inflammatory response, pulpal and periradicular tissue reactions, sealability,[5] *in vitro* fibroblastic stimulation and antimicrobial activity.[19] However, MTA-Angelus has a high pH and greater release of calcium in the first 24 h of activation,[14] possibly because of the greater amount of Portland cement or other calcium release agents[20] In the present report, white MTA-Angelus was used in one case and gray in the other with similar results.

The main disadvantages of Pro-Root MTA are poor hand ling characteristics due to granular sand-like consistency, looseness,[21] delayed setting time(2 h 45 min) and high cost. Long hardening time of ProRoot MTA requires a twovisit procedure for definitive treatment of the tooth. MTAAngelus does not contain calcium sulfate, which reduces its setting time to 10 min.[12] It has been shown that the small particle size would be an important aspect of a perforation repair material because it increases the surface available for hydration and enhances early strength. A recent study has shown that MTA-Angelus contains higher number of small particles than ProRoot MTA.[22] MTA-Angelus is found to have good handling characteristics in addition to faster setting time; further, it is cost-effective.[12]

One of the main goals of management of strip perforation is immediate repair of perforation to reduce the possibility of bacterial contamination as well as inflammatory process in the defect area for better post-treatment prognosis.[2,3] In the presented cases, time lapsed from the creation of perforation to the repair of the defect did not exceed 6 months. In case report 1, the option of immediately sealing the strip perforation in single visit provided for an adequate endodontic therapy that was free of hemorrhage and contamination, which would have otherwise negatively influenced the outcome of endodontic therapy. Control of hemorrhage was achieved with successive copious irrigation with 3% NaOCl, and therefore, sealing was accomplished while obturating the entire mesiobuccal canal with MTA-Angelus. To prevent overfilling or underfilling, a resorbable collagen matrix calcium phosphate can be applied before placing the MTA.[23] However, the use of matrix depends on the size of the lesion. However, Arens and Torabinejad[24] concluded in their study that MTA does not need a barrier. In the cases presented here, strip perforation was small with low risk of filling material extrusion. However, slight extrusion of the MTA was observed [Figure 2c], which was later replaced by hard tissue deposition.[25] In an animal study, when MTA accidentally extruded into the periradicular tissue, hard tissue deposition and cementum were observed over the materials along with a regeneration of periodontal tissues.

## CONCLUSION

The use of MTA has been reported for several different endodontic treatments; however, there is limited literature on its success as an obturating material in the treatment of strip perforation. Two cases of strip perforation have been described here. Case 1 suggests that MTA can be used as an alternative root canal obturation material for the treatment of strip perforation. In case 2, the location of the perforations at the level of the epithelial attachment and crestal bone suggested a guarded prognosis; MTA treatment was successful as indicated by follow up after 6 months and 2 years.
